# Ets2 knockdown inhibits tumorigenesis in esophageal squamous cell carcinoma *in vivo* and *in vitro*

**DOI:** 10.18632/oncotarget.11369

**Published:** 2016-08-18

**Authors:** Qinghua Li, Lu Yang, Kang Han, Liqiang Zhu, Yanting Zhang, Shanshan Ma, Kun Zhang, Bo Yang, Fangxia Guan

**Affiliations:** ^1^ The First Affiliated Hospital of Zhengzhou University, Zhengzhou 450052, Henan Province, China; ^2^ School of Life Sciences, Zhengzhou University, Zhengzhou 450001, Henan Province, China; ^3^ The Second Affiliated Hospital of Zhengzhou University, Zhengzhou 450002, Henan Province, China

**Keywords:** Ets2, esophageal squamous cell carcinoma, proliferation, apoptosis, mTOR/p70S6K signaling pathway

## Abstract

Increased expression of Ets2 is reported upregulated in esophageal squamous cell carcinoma tissue. However, the function of Ets2 in carcinogenesis of ESCC is poorly understood. Here, the rise of Ets2 was confirmed in ESCC cells and Ets2 depletion by RNA interference promotes cell apoptosis, inhibits cell proliferation, attenuates cell invasion and induces cell cycle G0/G1 arrest *in vitro*. Moreover, *in vivo*, Xenograft mouse model studies showed Ets2 knockdown inhibits tumor formation and metastasis significantly. Furthermore, Ets2 depletion inactivates the mTOR/p70S6K signaling pathway both *in vitro* and *in vivo*. Taken together, these findings strongly suggest that a critical role of Ets2 in human ESCC pathogenesis via the inactivation of the mTOR/p70S6K signaling pathway.

## INTRODUCTION

Esophageal squamous cell carcinoma (ESCC) is one of the most frequently diagnosed cancers in developing countries, especially in Northern China [1]. Although therapeutic strategies have improved, the prognosis of patients with ESCC is still poor owing to early invasion and metastasis and the 5-year survival rate of this disease after surgery and radiation therapy is only 10~20% [2]. The dismal outcome of ESCC is attributed to the largely unknown molecular mechanism in tumorigenesis and progression. In order to ultimately reduce morbidity and mortality of the disease, a greater understanding of the molecular events underlying carcinogenesis, progression and metastasis of ESCC is required.

The Ets was originally discovered as part of the gag-myb-ets transforming fusion protein of an avian replication-defective retrovirus, E26 [3], and the Ets family has been established as one of the largest families of transcriptional regulators, with diverse functions and activities. To date, 28 human Ets family members have been identified [3]. All the Ets transcription factors comprise a large evolutionarily conserved gene family characterized by the Ets domain, and this 85 amino acid region forms the winged helix-turn-helix DNA binding domain composed of three alpha helices and a four-stranded beta sheet. The Ets protein controls gene expression by binding to numerous genes with GGA (A/T) Ets binding site (EBS) [4] and over 400 Ets target genes have been defined based upon the presence of functional EBS in their regulatory regions [3], thus the Ets transcription factors impact a broad spectrum of cellular functions including proliferation, cell cycle, differentiation, migration, transformation, and apoptosis [5–8]. Ets2 is one of the founder members of the Ets family located on human chromosome 21 and was initially characterized as a proto-oncogene. Multiple studies with cell lines and animal models suggest that Ets2 causally exhibit both tumor-promoting and tumor-suppressive effects in distinct carcinomas. For example, Ets2 amplification has also been demonstrated in patients with acute nonlymphoblastic leukaemia [9], and Ets2 is expressed at elevated levels in breast, ocular neoplasm, cervical and prostate cancer [10–13]. However, Kabbout *et al*. revealed marked decline of Ets2 transcript expression in non-small cell lung cancer compared with paired normal lung tissues [14], exhibiting the tumor-suppressive effects of Ets2. The tumor-promoting/−suppressive effects of Ets2 might depend on the protein expression with potentially important functions in the tumor microenvironment, including growth factors, adhesion molecules, extracellular proteases and anti-apoptotic genes. Ets2 is a downstream target for both the Ras/Raf/MAP kinase and phosphatidylinositol 3-kinase/Akt pathways [15, 16], regulating the expression of a number of genes with potentially important functions in the tumor microenvironment.

Although Li *et al*. observed that Ets2 rose 75.7% (28/37) at mRNA level and 75% (12/16) at protein level in ESCC tissues relative to matched normal tissues [18], little work has been carried out at the molecular and cellular mechanisms of Ets2 action. Herein, we report on the results of Ets2 knockdown mediated by siRNA fragments and lentivirus on apoptosis, invasion, *et al*. and the signaling Ets2 involved in ESCC both *in vitro* and *in vivo*.

## RESULTS

### Overexpressed Ets2 is depleted in the ESCC cell lines

**Figure 1:**
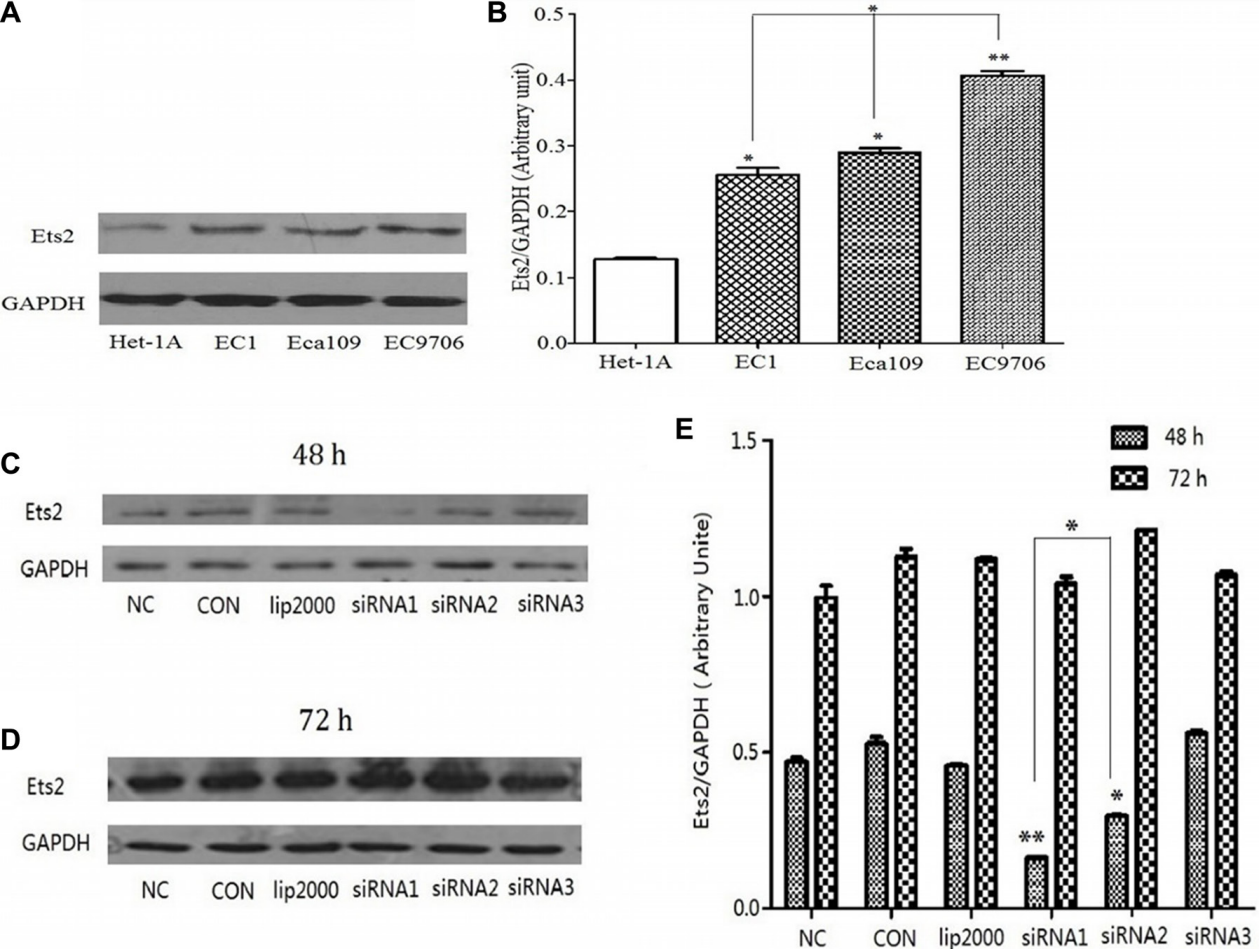
Expression of Ets2 protein was notably increased in ESCC cells and knocked down by siRNA (**A**) Western Blot analysis of Ets2 in Het-1A, EC1, EC9706 and Eca109 cells. (**B**) Semi-quantitative analysis showed that Ets2 was increased by 3.2-fold in EC9706 cells (*P* < 0.01), 2.2-fold in Eca109 cells (*P* < 0.05), and 1.93-fold in EC1 cells (*P* < 0.05) respectively compared with that in Het-1A cells. *P < 0.05, **P < 0.01 versus that in Het-1A cells. (**C**) and (**D**) Western blotting analysis of the expression of Ets2 in EC9706 cells at 48 h and 72 h after transfection with 3 candidate siRNA sequences (marked as siRNA1, siRNA2, siRNA3). (**E**) Quantitative results of the Western blotting analysis obtained via densitometric analysis. Ets2 protein expression was obviously inhibited by interference only with siRNA1 and siRNA2 (*P* < 0.01) sequences at 48 h after transfection and the interference efficiency of siRNA1 sequence was higher than that of siRNA2 (*P* < 0.05). *P < 0.05, **P < 0.01 versus the NC groups. CON, ESCC cells were cultured normally; lip2000, ESCC cells were transfected with equal amounts of Lipofectamine^®^ 2000 Reagent; NC, ESCC cell were transfected with non-targeting control siRNA as negative control.

Firstly, we confirmed whether Ets2 expression level was elevated in ESCC cells by Western blotting and the results demonstrated that the Ets2 expression was increased by 3.2-fold in EC9706 cells (*P* < 0.01), 2.2-fold in Eca109 cells (*P* < 0.05), and 1.93-fold in EC1 cells (*P* < 0.05) respectively compared with that in Het-1A cells (Figure 1A and 1B). And, the expression of Ets2 protein in EC9706 cells was higher than that in Eca109 and EC1 cells (*P* < 0.05).

To better understand the role of Ets2 in ESCC, three candidate siRNA fragments again Ets2 (marked as siRNA1, siRNA2, siRNA3) were synthesized to interfere Ets2 expression and the recombinant siRNA particles were transfected into ESCC cells with Lipofectamine^®^ 2000 Reagent (Invitrogen) following the manufacturer's protocol. ESCC cells transfected with equal amounts of Lipofectamine^®^ 2000 Reagent (lip2000) were used to eliminate the influence of the transfection reagent, ESCC cells were cultured as control (CON) and ESCC cell were transfected with non-targeting control siRNA as negative control (NC). As shown in Figure 1C and 1D, Ets2 was significantly decreased compared with NC and CON only at 48 h after transfection with siRNA1 and siRNA2 fragments in EC9706 cells. And as shown in Figure 1E, the interference efficiency of siRNA1 fragments was significantly higher than that of siRNA2 and siRNA3. Thus the siRNA1 sequence against Ets2 was chosen to knock Ets2 down and the optimal time for observation was at 48 h after transfection.

### Ets2 knockdown suppresses ESCC cells proliferation *in vitro* and *in vivo*

**Figure 2:**
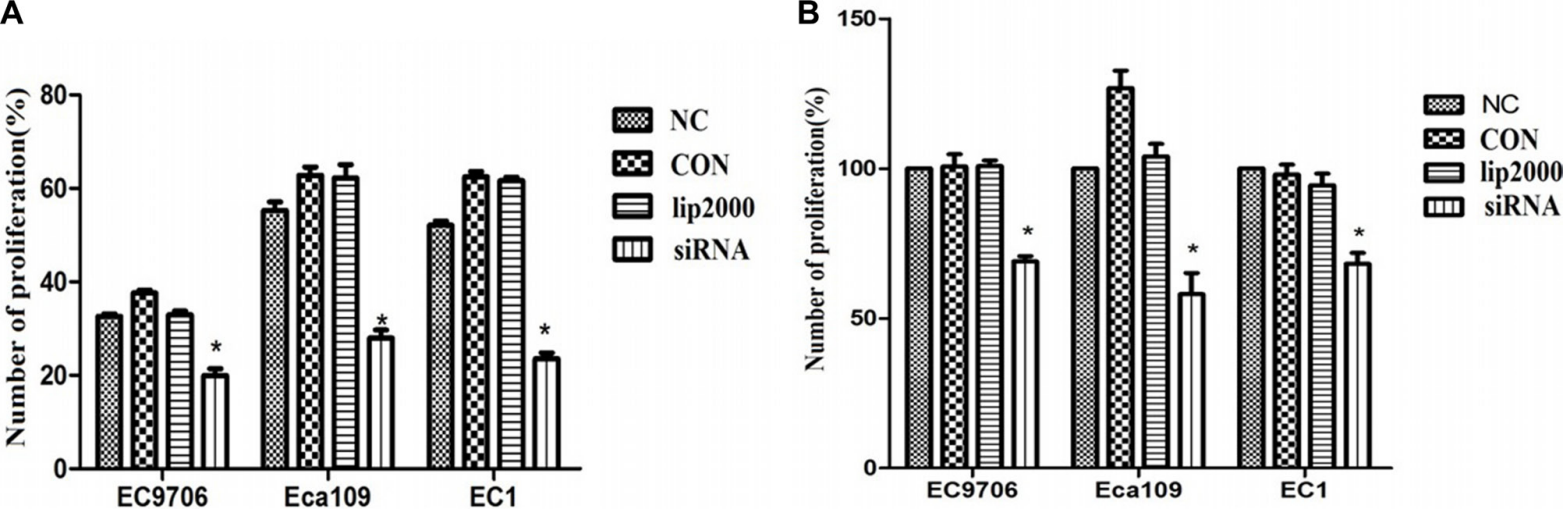
Proliferative rates of ESCC cells when Ets2 was knocked down (**A**) the numbers of proliferative rates of siEts2-EC9706, siEts2-Eca109 and siEts2-EC1 cells were 19.97%, 29.2% and 22.3% respectively, reducing by 40%, 45% and 57% compared with NC cells measured with EdU assay. (**B**) the cell survival rates of EC9706, Eca109 and EC1 cells were 69%, 58% and 68% after transfection with siEts2, reducing by 31%, 42% and 32% compared with NC (Figure 3B), respectively. *P < 0.05.

We next investigated the effects of Ets2 depletion on cell proliferation and survival by 5-ethynyl-2′-deoxyuridine (EdU) assay and CCK-8. The Edu assay revealed that the proliferative rates of EC9706, Eca109 and EC1 cells worked out 19.97%, 29.2% and 22.3% respectively after Ets2 depletion, reducing by 40%, 45% and 57% compared with NC cells (Figure 2A). Meanwhile, detection of cell viability of ESCC cells by CCK-8 showed that the cell survival rates of EC9706, Eca109 and EC1 cells were 69%, 58% and 68% after transfection with siEts2, reducing by 31%, 42% and 32% compared with NC (Figure 2B), respectively. Additionally, the proliferative rates and cell survival rates changed without differences among NC, CON and lip2000 groups. These data suggest that the Ets2 decrease could suppress the proliferation of ESCC cells *in vitro*.

In order to confirm whether the growth-inhibiting effect of Ets2 depletion is relevant to ESCC growth *in vivo*, silent expression of Ets2 were inoculated into BALB/C athymic mice after siRNA1 sequence was ligated into retroviral vectors to establish the Eca109 cell stably silencing Ets2. Silencing Ets2 expression inhibited tumor growth (Figure 7A) and induced a decrease in tumor weight and volume (Figure 7B). Taking all together, we concluded that Ets2 inhibits the proliferation of ESCC cells *in vivo* and *in vitro*.

### Ets2 depletion promotes apoptosis of ESCC cells *in vitro* and *in vivo*

**Figure 3:**
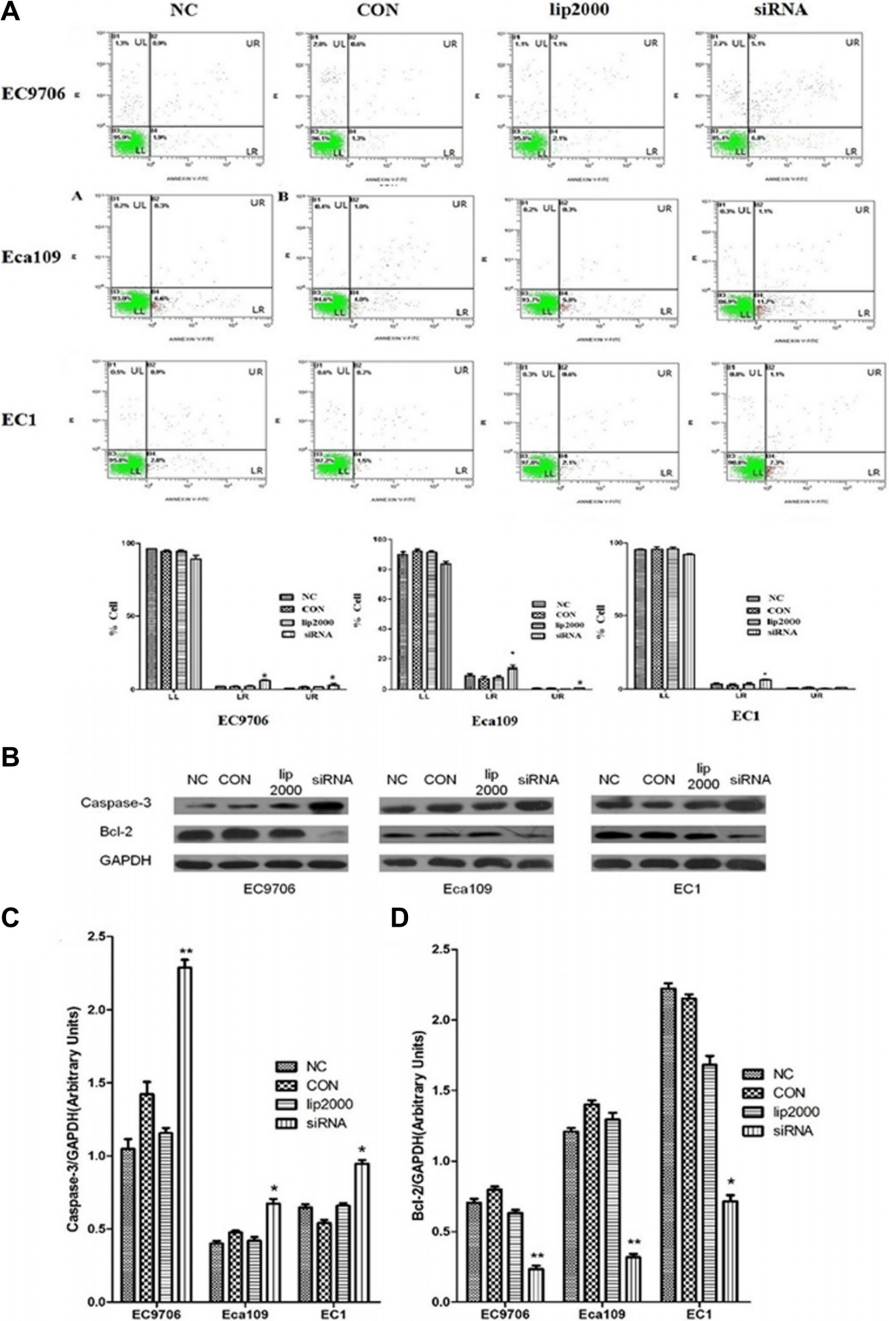
Ets2 knockdown induced apoptosis of ESCC cells *in vitro* (**A**) apoptosis of ESCC cells was detected using annexin V-FITC and PI dual staining. Quadrants were designed as follows, LL: non-stained cells indicating viable cells; LR: annexin V-FITC stained cells indicating early apoptosis; UR: annexin V-FITC and PI stained cells indicating late apoptosis; and UL: PI stained cells indicating secondary necrosis. All dot plots are a representation of equal cell populations (*n* = 10,000). (**B**) apoptosis factor proteins caspase-3 and Bcl-2 were analyzed by Western blotting. (**C**) and (**D**) semi-quantitative analysis of caspase-3 and Bcl-2 expression. Caspase-3 was increased by 120%, 75% and 30% roughly in EC9706, Eca109 and EC1 cells compared with NC, while anti-apoptotic Bcl-2 protein was decreased by 70%, 73% and 68% in EC9706, Eca109 and EC1 cells compared with NC. *P < 0.05, **P < 0.01.

Apoptotic cell death of ESCC cells was examined using flow cytometric analysis staining with FITC-conjugated annexin-V as an early marker for apoptosis. Cells were simultaneously stained with PI to investigate loss of cell membrane integrity. This double staining procedure distinguishes early stage apoptotic cells (annexin V-positive) from late stage apoptotic cells (annexin V-positive, PI positive). As shown in Figure 3A, when Ets2 was knocked down in EC9706 cells, the percentages of early and late stage apoptotic cells were increased from 1.9% and 0.9% in NC to 6.8% and 5.1% respectively (*P* < 0.05). Early and late stage apoptotic cells in Eca109 cells were increased from 6.6% in NC to 11.7% and from 0.3% in NC to 1.1% respectively (*P* < 0.05). However, only the late stage apoptotic cells were increased from 2.8% to 7.3% (*P* < 0.05) in Ets2-depleted EC1 cells. Furthermore, Tunel assay showed that apoptotic rate was 17.4% in Ets2 depleted tumor tissue (Figure 8).

In addition, the cell apoptosis factor caspase-3 and Bcl-2 were detected in our study *in vitro* and *in vivo*. The results of Western blotting revealed cell apoptosis factor caspase-3 was increased dramatically compared to their corresponding control cells and tissue (Figure 3B and D, *P* < 0.05). In contrast, anti-apoptotic Bcl-2 protein was decreased when Ets2 was knocked-down (Figure 7C and 7D, *P* < 0.001). Taken together, these results indicated that early stage apoptosis was induced by depletion of Ets2 in ESCC cells *in vitro* and *in vivo.*

### Ets2 depletion induces G0/G1 period arrest in Eca109 and EC1 cells *in vitro*

**Figure 4:**
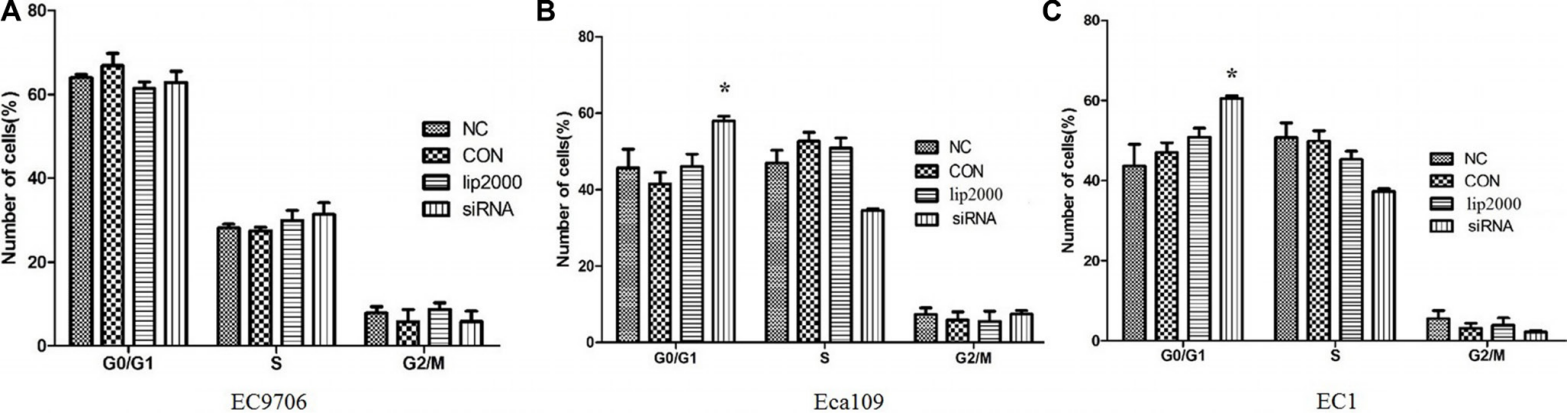
Effects of Ets2 knockdown on cell cycle The number of siEts2-EC9706 cells at each cell cycle phase had no change compared with that of NC cells. The numbers of siEts2-Eca109 and siEts2-EC1 cells at G0/G1 phase were more than that of CON cells (*P* < 0.05). *P < 0.05.

To explore the effect of Ets2 on cell cycle of ESCC cells, the cell cycle of ESCC cells were measured using flow cytometry after treatment with siRNA. FACScan analysis of PI-stained cells revealed that there were no significant change in cell cycle distribution of EC9706 cells compared with that in NC cells (Figure 4A, *P* > 0.05). Nevertheless, more cells accumulated in the G0/G1 phase of the cell cycle in Eca109 and EC1 cells compared with that in NC cells (Figure 4B and 4C, *P* < 0.05) when Ets2 was depletion, indicating Ets2 knockdown could arrest Eca109 and EC1 cells at G0/G1 phase.

### Ets2 depletion inhibits ESCC cells invasion

**Figure 5:**
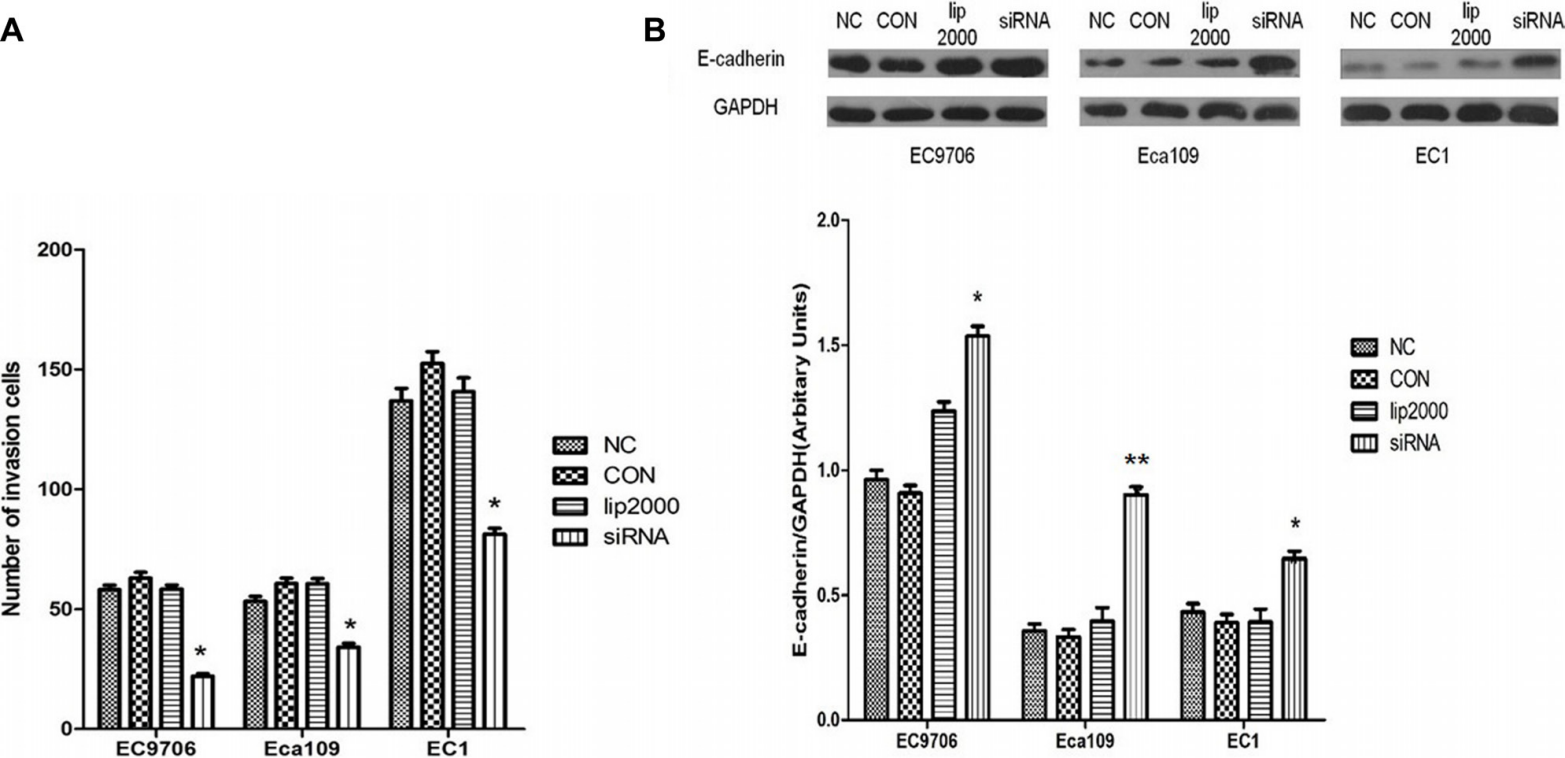
Inhibitory effects of Ets2 depletion on the invasive capacity of ESCC cells (**A**) The results of Transwell chamber assay. The numbers of invaded siEts2-EC9706, siEts2-Eca109 and siEts2-EC1 cells were significantly reduced compared with that of invaded NC cells. (**B**) E-cadherin expression was detected using Western blotting and the results revealed that E-cadherin protein was significantly increased by 54%, 130% and 67% respectively compared with that in NC. *P < 0.05, **P < 0.01.

We also investigated whether Ets2 affects the invasive ability of ESCC cells by an *in vitro* invasion assay. As shown in Figure 5A, knockdown of Ets2 extremely reduced the cell invasive ability. When Ets2 was interfered, the number of invaded EC9706 cells was 23.22 ± 3.03 compared with 58.66 ± 5.74 of NC cells (*P* < 0.05), 35.33 ± 1.53 compared with 54 ± 3.61 of NC (*P* < 0.05) of Eca109 cells and 81.33 ± 5.85compared with 137±1.26 of NC (*P* < 0.05) of EC1 cells. We also examined the expression of cellular adhesion molecule, E-cadherin by western blotting *in vitro* and *in vivo*, finding that E-cadherin proteins in EC9706, Eca109 and EC1 cells rose by 54%, 130% and 67% respectively compared with that in NC (Figure 5B, *P* < 0.05). Moreover, E-cadherin was also increased in Ets2-knockdown tumor tissues compared to tumor-bearing control tissues (Figure 7C and 7D, *P* < 0.001). These data suggested that Ets2 silence was related to invasion inhibition of ESCC cells.

### Ets2 knockdown inactivates the mTOR/p70S6K signaling pathway *in vitro* and *in vivo*

**Figure 6:**
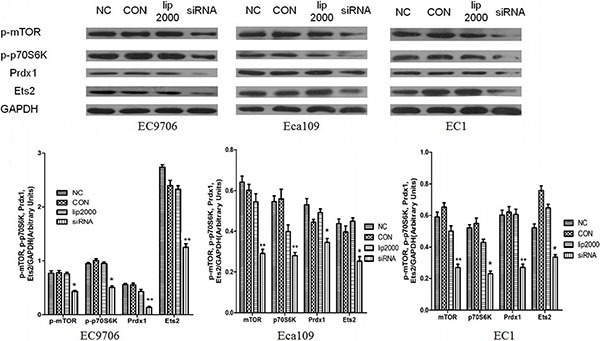
Effect of Ets2 knockdown on mTOR/p70S6K signaling pathway When Ets2 was knocked down, the expression of Prdx1 protein was reduced concomitantly with the reduction of Ets2 in EC9706, Eca109 and EC1 cells (*P* < 0.05). Both p-mTOR and p-p70S6K proteins were decreased in siEts2-EC9706, siEts2-Eca109 and siEts2-EC1 cells compared with that in NC groups (*P* < 0.05). *P < 0.05, **P < 0.01.

**Figure 7:**
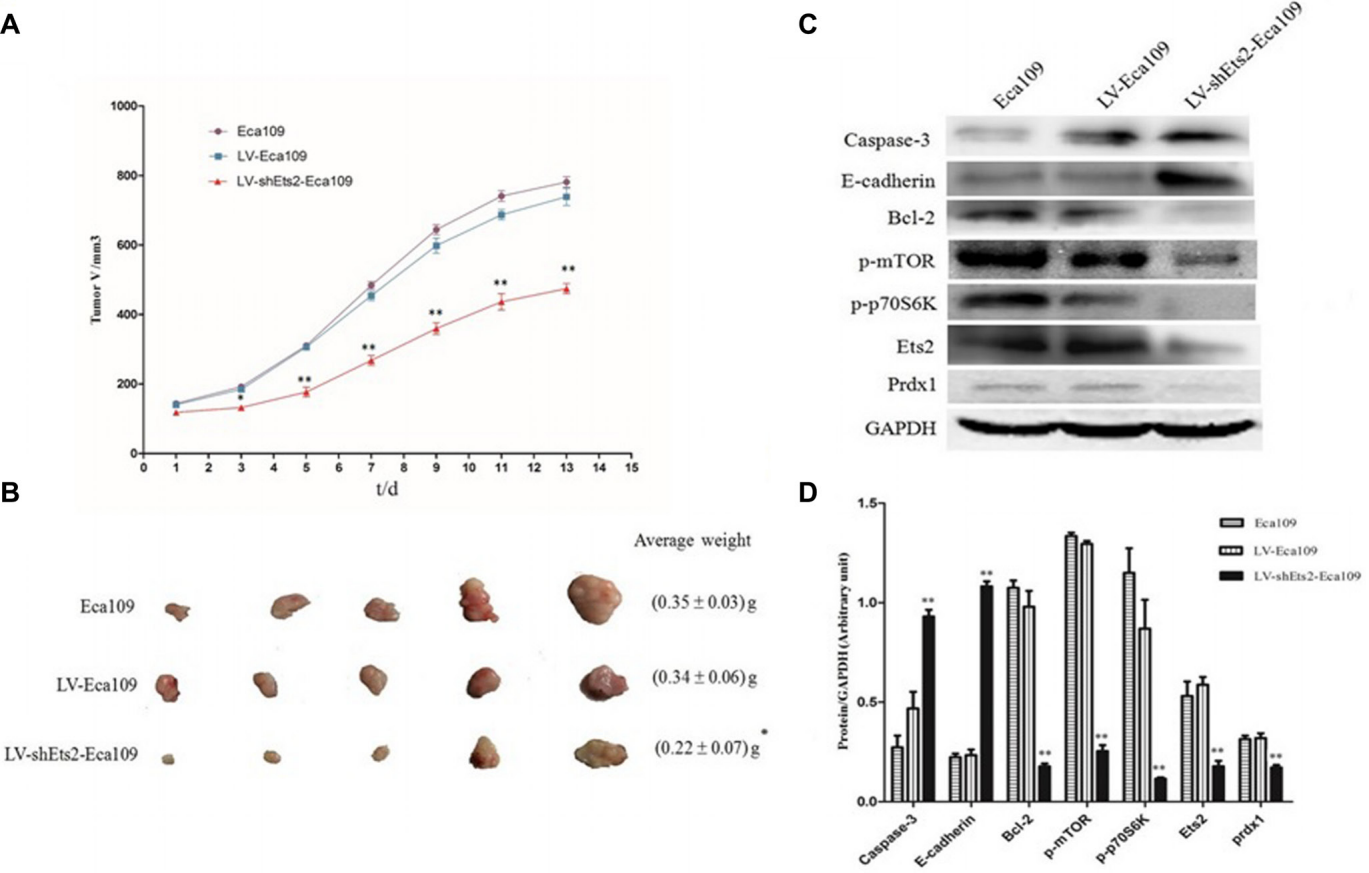
Effects of Ets2 silence on tumor size and protein expressions *in vivo* (**A**) the tumor growth curve of xenograft mice. The tumor volume in LV-shEts2-Eca109 cell injected into nude mice was significantly smaller than that in LV-Eca109 and Eca109 cell-bearing mice (*P* < 0.001). (**B**) the tumor weight in xenograft mice. The average tumor weight in LV-shEts2-Eca109 cell-bearing mice was much lighter than that in LV-Eca109 and Eca109 cell-bearing mice (*P* < 0.05). (**C**) and (**D**), the protein expressions were analyzed by Western blotting. Protein caspase-3 and E-cadherin were significantly enhanced in LV-shEts2-Eca109 injected mice compared with that in Eca109 and LV-Eca109 injected mice (*P* < 0.001), while the proteins of Bcl-2, p-mTOR, p-p70S6K and Prdx1 were significantly reduced as the reduction of Ets2 in xenograft mice (*P* < 0.001). *P < 0.05, **P < 0.01.

To examine the mechanistic basis of Ets2 in ESCC, we determined the effects of Ets2 knockdown on the expression of phosphorylated mTOR (p-mTOR), p70S6K and Prdx1 of ESCC cell *in vitro* and cancer xenograft mouse model. Western blotting analysis showed that siRNA-mediated knockdown of Ets2 decreased the expression of p-mTOR and p-p70S6K dramatically in EC9706 Eca109 and EC1 cells and Eca109-bearing tissues as shown in Figures 6, 7C and 7D, indicating that Ets2 knockdown inactivated the mTOR/p70S6K signaling pathway remarkably. And Peroxiredoxin 1 (Prdx1) which was reported associated with Ets2 was also reduced by Ets2 knockdown in ESCC cells (Figure 7C and 7D, *P* < 0.001).

## DISCUSSION

Cancer can be defined as a genetic disease, resulting as a consequence of multiple events associated with initiation, promotion and metastatic growth. Ets proteins are transcription factors that activate or repress the expression of genes, which are involved in various biological processes, including cellular proliferation, differentiation, development, transformation and apoptosis [3]. Ets2 was originally identified as a proto-oncogene, but it exhibits both tumor-promoting and -suppressive effect in different types of carcinomas. Previous studies have indicated that Ets2 factors are abnormally expressed in both tumor and stromal compartments, which frequently correlates with tumor progression. For example, Ets2 are expressed at much higher levels in neoplasias of the thyroid relative to benign and normal tissues [20]. A high Ets2 protein level in early stage of hepatocellular carcinoma has also been found [21]. Expression of Ets2 is not observed in normal colon and hyperplastic polyps, however its expression is associated with advancing tumor grade and correlates with lymph node metastasis in colon cancer [17], and Ets2 deregulated in non-small cell lung cancer was identified as one of 50 genes in mice that are involved in the progression of lung cancer from adenoma to carcinoma [22]. Previously, it was demonstrated that Ets2 expression was enhanced in ESCC tissues [18]. In the present study, we further corroborated that Ets2 was also expressed at a relatively higher level in ESCC cells compared to normal esophageal squamous cells. To elucidate the mechanism basis of contribution of Ets2 to the development and progression of ESCC, we constructed an Ets2-knockdown cell model mediated by RNA interference. The most effective siRNA sequence was screened from 3 candidate sequences and Ets2 protein was specifically inhibited in ESCC cell lines. When Ets2 was knocked down by RNA interference, the viability and proliferation rates of ESCC cells detected by CCK-8 and EdU assays were distinctly decreased *in vitro*. Ets2 depletion enhanced early and late stage apoptosis in EC9706 and Eca109 cells but only promoted early stage apoptosis in EC1 cells. The cell apoptosis factor caspase-3 was increased while anti-apoptotic Bcl-2 protein was decreased corresponding with the decrease in Ets2 levels. These observations suggest the presence of an intrinsic cell death pathway stimulating a wide network of signals which acts in part through the Bcl-2 family signaling cascade [23]. We also found G0/G1 period arrest in Eca109 and EC1 cells induced by Ets2 knockdown *in vitro*. The number of ESCC cells that invaded Transwell chambers pre-coated with matrigel was significantly reduces and the expression of cellular adhesion molecule E-cadherin which mediates homophilic calcium dependent intracellular adhesion was enhanced after a reduction of Ets2 *in vitro* and *in vivo*, illustrating Ets2 knockdown inhibited the invasion of ESCC cells via strengthen the cellular adhesion.

**Figure 8:**
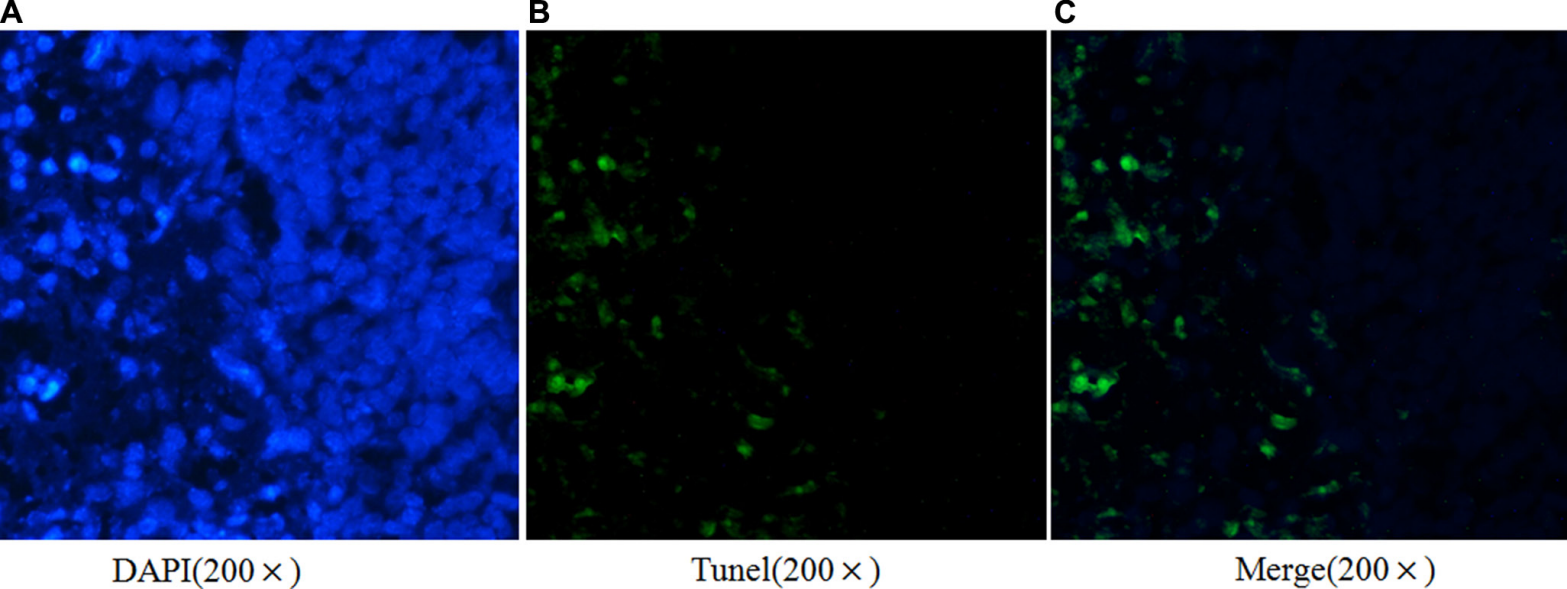
Ets2 silence promotes ESCC cells apoptosis *in vivo* Tunel assay demonstrated a 17.4% apoptotic rate in Ets2 silence tissue.

Ets proteins contribute to many biological processes by control and interaction of specific target genes in various cancers. Ets2 are demonstrated to be involved in both Ras/Raf/MAP kinase and phosphatidylinositol 3-kinase/Akt pathways in murine fibroblasts. Additionally, Ets2 has been identified as a Wnt target in colorectal cancer cells and intestinal stem cells [17]. The mammalian target of rapamycin (mTOR) is a serine/threonine protein kinase that supports cell growth, cellular metabolism, cell proliferation, cell motility, cell survival, protein synthesis, and transcription initiating angiogenesis and autophagy. Our previous study found that the mTOR/p70S6K signaling pathway was aberrantly activated in ESCC cells [24] and that the activity of mTOR/p70S6K signaling pathway was decreased when Prdx1, a tumor-associated antigen in ESCC [25], was reduced in EC9706 cells [26]. In addition, Masaki et al. found that Ets-2 regulated the expression of Prdx1 in human prostate cancer PC3 cells [27]. Thus, we hypothesized that Ets2 could be involved in ESCC tumorigenesis through mTOR/p70S6K signaling pathway. In our study, we found p-mTOR and p-p70S6K proteins significantly reduced and this was accompanied by the knockdown of Ets2 *in vitro* and *in vivo*, indicating that Ets2 knockdown inactivated the mTOR/p70S6K signaling pathway.

Collectively, Ets2 knockdown by RNA interference effectively promoted ESCC cell apoptosis, decreased ESCC cell proliferation, inhibited migration, induced G0/G1 arrest and attenuated invasion of ESCC cells *in vitro* and *in vivo*, via inactivating the mTOR/p70S6K signaling pathway. The finding in this study could provide novel therapeutic targets and diagnostics for future ESCC therapy, as direct inhibition of Ets2 transcription by triplex forming oligonucleotides resulted in growth inhibition and induction of apoptosis in human prostate cancer cells [28].

## MATERIALS AND METHODS

### Cell lines and cell culture

ESCC cell lines EC1 and Eca109 were preserved in our laboratory and EC9706 cells were provided by the State Key Laboratory of Molecular Oncology, Chinese Academy of Medical Sciences (Beijing, China). An immortalized normal esophageal epithelial cell line Het-1A was a gift of Prof. Zhenyu Ji (Henan Academy of Medical and Pharmaceutical Sciences, China). STR analysis was performed to ensure the cells are tumor cells (Supplementary material). Cells were cultured in RPMI 1640 medium (HyClone Laboratories, USA) supplemented with 10% fetal bovine serum (FBS) (TBD Science, China), at 37°C in the presence of 5% CO2.

### Ets2 knockdown by RNA interference

Three candidate siRNA targeting sequences against Ets2 (siEts2) were synthesized by Shanghai GenePharma Co., Ltd (Shanghai, China) after our design. The silencing effect of the siRNA was then evaluated by Western blotting. For assays *in vivo*, the most effective target sequence was ligated into lentiviral pFH-L plasmid (Shanghai Genechem), packaged in HEK-293T cells and then lentivirus particles of Ets2 (LV-shEts2) were collected with lentivirus particles of scramble shRNA as a negative control (LV). Then, lentivirus particles of siEts2 and scramble shRNA were added to the culture medium to infect Eca109 cells (marked as LV-shEts2-Eca109 and LV-Eca109). After lentivirus infection, the cells were washed with PBS and collected to inject into athymic nude mice.

### Western blotting analysis

The total proteins of ESCC cells and tissues were extracted with Total Protein Extraction (Sangon Biotech, China). The protein extracts were separated with SDS-PAGE after their concentrations were determined using Bradford protein assay kit (Sangon Biotech, China) and then transferred to polyvinylidine difluoride microporous (PVDF) membranes (Millipore, USA). The blotted membranes were treated with 5% (w/v) skimmed milk in TBST buffer (10 mmol/L Tris, 150 mmol/L NaCl, and 0.2% Tween 20), and incubated for 1 h at room temperature with primary antibody anti Ets2 (Santa Cruz Biotechnology, USA), p-p70S6K (Cell Signaling Technology, USA), Peroxiredoxin 1 (Cell Signaling Technology, USA) and caspase-3 (Abcam, USA), E-cadherin (Abcam, USA) and GAPDH (Cell Signaling Technology, USA). They were then washed and incubated for 1 h with a horseradish peroxidase-linked anti-rabbit secondary antibody (Sangon Biotech, China). Finally, the bands of specific proteins on the membranes were examined by Enhanced Chemiluminescence Kit (Beijing ComWin Biotech Co., Ltd, China) and densitometric analysis was performed with Image J software (NIH).

### Cell proliferation assay *in vitro*

Cell proliferation was measured by the 5-ethynyl-2′-deoxyuridine (EdU) assay according to the manufacturer's instructions. Briefly, 5×10^5^ cells/well were seeded in a 96-well flat-bottomed plate, grown at 37°C for 24 h, and then exposed to 50 μM of EdU for additional 2 h at 37°C. Cells were fixed with 4% formaldehyde for 15 min and permeabilized with 0.5% Triton X-100 for 20 min. After being rinsed three times with PBS, the cells were treated with ApolloR reaction cocktail followed by DNA staining with Hoechst 33342 for 30 min for visualizing under fluorescent microscope (Nikon, Japan).

### Cell apoptosis assay *in vitro* and *in vivo*

Apoptotic cells were determined with an Annexin V-FITC cell apoptosis kit (BD Biosciences, USA) according to the manufacturer's protocol. Briefly, the transfected ESCC cells were washed with PBS and subsequently incubated for 15 min at room temperature in the dark in 100 μl 1 × binding buffer containing 5 μl Annexin V-FITC and 5 μl propidiumiodide (PI). Apoptosis was determined by fluorescence microscope (Zeiss Axio Observer A) and FACScan laser flow cytometer (FACSCalibur, Becton Dickinson, USA).

We used the *In situ* cell death detection kit according to the manufacturer's instructions for the TUNEL assay. Briefly, 5 μm-paraffin sections were made from the center of the paraffin-embedded tumor tissues, and the sections were deparaffinized, washed with xylene, ethanol and PBS. Each section was incubated with 20 μg/mL proteinase K solution at 37°C for 30 min after permeation in 1% triton X-100 and blocking in 3% H_2_O_2_. TUNEL staining was performed using the Dead End fluorometric TUNEL system (KeyGEN BioTECH, China) according to the manufacturer's protocol.

### Transwell chamber assay for invasion *in vitro*

Transwell chambers pre-coated with matrigel (Corning-Costar, USA) were used to detect invasion capacity of siRNA-transfected EC9706, Eca109 and EC1 cells. Firstly, serum-free RPMI 1640 medium was planted into the upper of a 24-well Transwell plate with polycarbonate membrane filters containing 8-μm pores and then Transwell plate was incubated for 1.5 h in the cell incubator hydrating the extracellular matrix (ECM) membrane. After transfection for 48 h, the 300 μl cell suspension with 2 × 10^5^ cells /ml were planted into the upper compartment and the lower compartment of the Transwell plate contained 500 μl of RPMI1640 medium with 5% FBS. After an 8 h-incubation at 37°C, non-invading cells were gently removed using a cotton-tipped swab. The cells were then fixed with methanol for 10 min followed by 0.2% crystal violet staining. The number of invaded cells in 10 distinct randomly selected fields was counted under light microscope.

### *In vitro* cell cycle distribution analysis

EC9706, Eca109, and EC1 cells transfected with siRNA and control cells were respectively harvested, fixed in 70% ice-cold ethanol overnight at 4°C, and then stained with PI. Cell cycle analysis was carried out by flow cytometry (BD, USA). Establishment of Eca109 Cell Xenograft Mouse model *In Vivo*

A total of 15 pathogen-free male BALB/C athymic nude mice were purchased from VITAL RIVEA Laboratory Animal Co. Limited (Beijing, China). The 6-week old mice were divided into 3 groups after acclimatization for 7 days, each group was injected with 2 × 10^6^ Eca109, LV-shEts2-Eca109 and LV-Eca109 cells, respectively. After 7 days, the tumor length (long axis) and tumor width (short axis) of each tumor-bearing mouse were measured every other day for 2 weeks, and tumor volumes were calculated as 1/2 × length × width^2^ (mm^3^) according to previous method [19]. Mice were then sacrificed under deep anesthesia and the final volumes, as well as the weights of the tumors were measured. The tumor inhibition rate was calculated as (tumor weight in control mice-tumor weight in treated mice)/tumor weight in control mice × 100. Eventually, the tumor tissues were kept in liquid nitrogen for further investigations.

### Statistical analysis

All experiments were carried out in triplicate, and the experimental results were analyzed using a SPSS software package (version 17.0). All numerical data were expressed as means ±standard deviation (SD). ANOVA was used for multiple comparisons with a value of *P* < 0.05 considered to be statistically significant. All *P* values were generated from two-sided tests for statistical significance.

## SUPPLEMENTARY MATERIALS FIGURES AND TABLES










