# Supplementary material for: Ets2 knockdown inhibits tumorigenesis in esophageal squamous cell carcinoma in vivo and in vitro
Source: Oncotarget. 2016 Aug 18;7(38):61458–68. doi: 10.18632/oncotarget.11369 (PMC5308664; doi:10.18632/oncotarget.11369)
Supplement: Supplementary file 2 [file oncotarget-07-61458-s002.pdf]

GENEWIZ®

Solid science. Superior service.

Cell Line Authentication Report

GENEWIZ,Inc. Beijing

30 Science Park Road

Zhong-Guan-Cun Life Science Park

Changping District, 102206

Beijing, China

Tel: 400-8100-669

Fax: 010-59458058

Email: Genomics.China@genewiz.com.cn

www.genewiz.com.cn

GENEWIZ,Inc. Beijing
30 Life Science Park Road
Changping District, 102206
Beijing, China
Tel: 400-8100-669
Fax: 010-59458058
www.genewiz.com.cn

Cell Line Authentication Report

Customer: LiQingHua

Institution: Zhengzhou University

Quotation Number: HJ1603221

Completion Date：03/31/2016

1. **Sample ID: EC109**
2. **Original Material: Cell pellets**
3. **Methods:**

1).Genomic DNA was extracted from the cell pellets provided by the customer.

2).Samples, together with positive and negative control were amplified using GenePrint 10 System (Promega).

3). Amplified products were processed using the ABI3730xl Genetic Analyzer.

4).Data were analyzed using GeneMapper4.0 software and then compared with the ATCC, DSMZ or JCRB databases for reference matching.

4. **Results:**

1) **10 Loci STR Profile:**

Genetic Site	Customer sample	
(Locus)	EC109	
Amelogenin	X	
CSF1PO	9	10
D13S317	12	13.3
D16S539	9	10
D5S818	11	12
D7S820	8	12
THO1	7	
TPOX	8	12
vWA	16	18
D21S11	27	28

GENEWIZ,Inc. Beijing
30 Life Science Park Road
Changping District, 102206
Beijing, China
Tel: 400-8100-669
Fax: 010-59458058
www.genewiz.com.cn

<<<If the Percent match is not 100%, search for reference matchingwith the ATCC, DSMZ or JCRB databases and add the match results.

Addendum: Comparative output from the ATCC STR Profile database

Result of STR matching analysis by your data.

- DSMZ Profile Database -

A graphical presentation is shown at the bottom of this page.

EV	Cell No.	Cell name	Locus names									Figures
			D5S818	D13S317	D7S820	D16S539	VWA	TH01	AM	TPOX	CSF1PO	
	Query (Your Cell)		11, 12	12, 13. 3	8, 12	9, 10	16, 18	7	X	8, 12	9, 10	
1.06(36/34)	57	HELA	11, 12	12, 13. 3	8, 12	9, 10	16, 18	7, 7	X, X	8, 12	9, 10	-
1.06(36/34)	57	HELA	11, 12	12, 13. 3	8, 12	9, 10	16, 18	7, 7	X, X	8, 12	9, 10	-
1.06(36/34)	116	GIRARDI HEART C2	11, 12	12, 13. 3	8, 12	9, 10	16, 18	7, 7	X, X	8, 12	9, 10	-
1.06(36/34)	121	GIRARDI HEART C7	11, 12	12, 13. 3	8, 12	9, 10	16, 18	7, 7	X, X	8, 12	9, 10	-
1.06(36/34)	136	KB	11, 12	12, 13. 3	8, 12	9, 10	16, 18	7, 7	X, X	8, 12	9, 10	-
1.06(36/34)	149	KB-V1	11, 12	12, 13. 3	8, 12	9, 10	16, 18	7, 7	X, X	8, 12	9, 10	-
1.06(36/34)	158	KB-3-1	11, 12	12, 13. 3	8, 12	9, 10	16, 18	7, 7	X, X	8, 12	9, 10	-
1.06(36/34)	161	HELA-S3	11, 12	12, 13. 3	8, 12	9, 10	16, 18	7, 7	X, X	8, 12	9, 10	-
1.06(36/34)	227	BT-B	11, 12	13. 3, 13. 3	8, 12	9, 10	16, 18	7, 7	X, X	8, 12	9, 10	-
1.06(36/34)	228	SBC-2	11, 12	12, 13. 3	12, 12	9, 10	16, 18	7, 7	X, X	8, 12	9, 10	-
1.06(36/34)	229	SBC-7	11, 12	12, 13. 3	12, 12	9, 10	16, 18	7, 7	X, X	8, 12	9, 10	-
1.06(36/34)	CCL-13	HeLa [Chang Liver]	12, 12	12, 13. 3	8, 12	9, 10	16, 18	7, 7	X, X	8, 12	10, 10	-

>>>

2) Electrophoretogram

GENEWIZ,Inc. Beijing
30 Life Science Park Road
Changping District, 102206
Beijing, China
Tel: 400-8100-669
Fax: 010-59458058
www.genewiz.com.cn

Applied Biosystems

M10023-1

GeneMapper 4.0

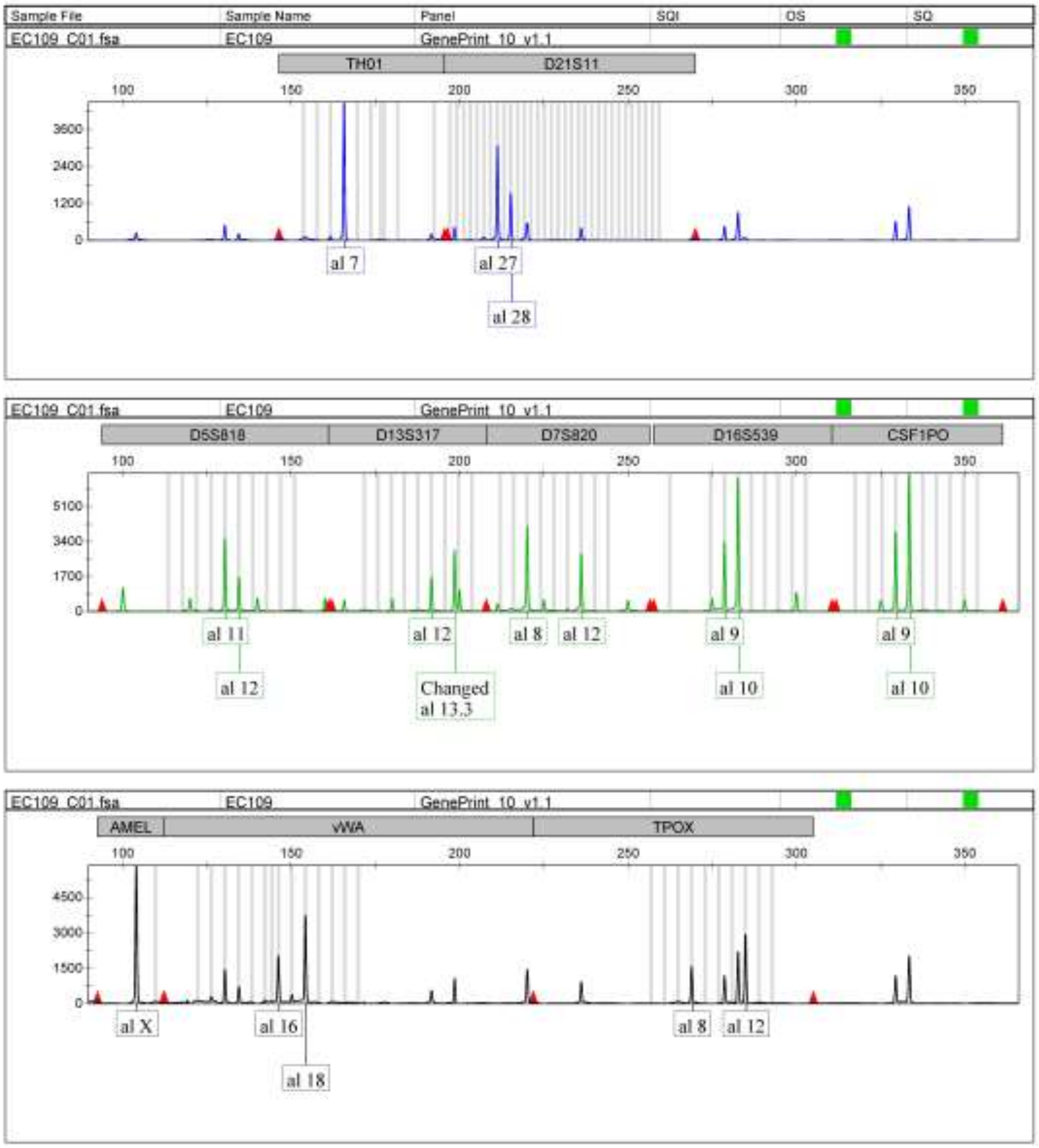

Thu Mar 31,2016 12:00PM, CST

Printed by: gm

Page 1 of 1

Note：Raw data in appendix
