# Supplementary material for: Ets2 knockdown inhibits tumorigenesis in esophageal squamous cell carcinoma in vivo and in vitro
Source: Oncotarget. 2016 Aug 18;7(38):61458–68. doi: 10.18632/oncotarget.11369 (PMC5308664; doi:10.18632/oncotarget.11369)
Supplement: Supplementary file 4 [file oncotarget-07-61458-s004.pdf]

GENEWIZ®
Solid science. Superior service.

Cell Line Authentication Report

GENEWIZ,Inc. Beijing

30 Science Park Road

Zhong-Guan-Cun Life Science Park

Changping District, 102206

Beijing, China

Tel: 400-8100-669

Fax: 010-59458058

Email: Genomics.China@genewiz.com.cn

www.genewiz.com.cn

GENEWIZ,Inc. Beijing
30 Life Science Park Road
Changping District, 102206
Beijing, China
Tel: 400-8100-669
Fax: 010-59458058
www.genewiz.com.cn

Cell Line Authentication Report

Customer: LiQingHua

Institution: Zhengzhou University

Quotation Number: HJ1603221

Completion Date：03/31/2016

1. **Sample ID: HET-1A**
2. **Original Material: Cell pellets**
3. **Methods:**

1).Genomic DNA was extracted from the cell pellets provided by the customer.

2).Samples, together with positive and negative control were amplified using GenePrint 10 System (Promega).

3). Amplified products were processed using the ABI3730xl Genetic Analyzer.

4).Data were analyzed using GeneMapper4.0 software and then compared with the ATCC, DSMZ or JCRB databases for reference matching.

4. **Results:**

1) 10 Loci STR Profile:

Genetic Site	ATCC		Customer sample	
(Locus)	HET-1A		HET-1A	
Amelogenin	X	Y	X	Y
CSF1PO	10	12	10	12
D13S317	11		11	
D16S539	9	11	9	11
D5S818	11	12	11	12
D7S820	9		9	
THO1	7		7	
TPOX	11		11	
vWA	16		16	
D21S11			28	31
Percent match between the sample and the database profile：100%				

GENEWIZ,Inc. Beijing
30 Life Science Park Road
Changping District, 102206
Beijing, China
Tel: 400-8100-669
Fax: 010-59458058
www.genewiz.com.cn

Summary:

1) Your cell line is considered to be "identical" tothe reference cell line in the ATCC STR database, as the STR profile yields a 100% match.

Notes：

1. P=100% x (2xM)/N; M=13, N=26 P=100% x (2x13)/26=100%

M: number of the matching peaks; N: number of all peaks

2. Based on the ANSI Standard, cell lines with ≥80% match are considered to be related; i.e.,derived from a common ancestry. Cell lines with between a 55% to 80% match require futher profiling for authentication of relatedness.

3. The short tandem repeat (STR) profile generated by GENEWIZ Inc. is indicative only of the sample sent to GENEWIZ Inc. at the time it was sent. This data and analysis are for research use only.

2) Electrophoretogram

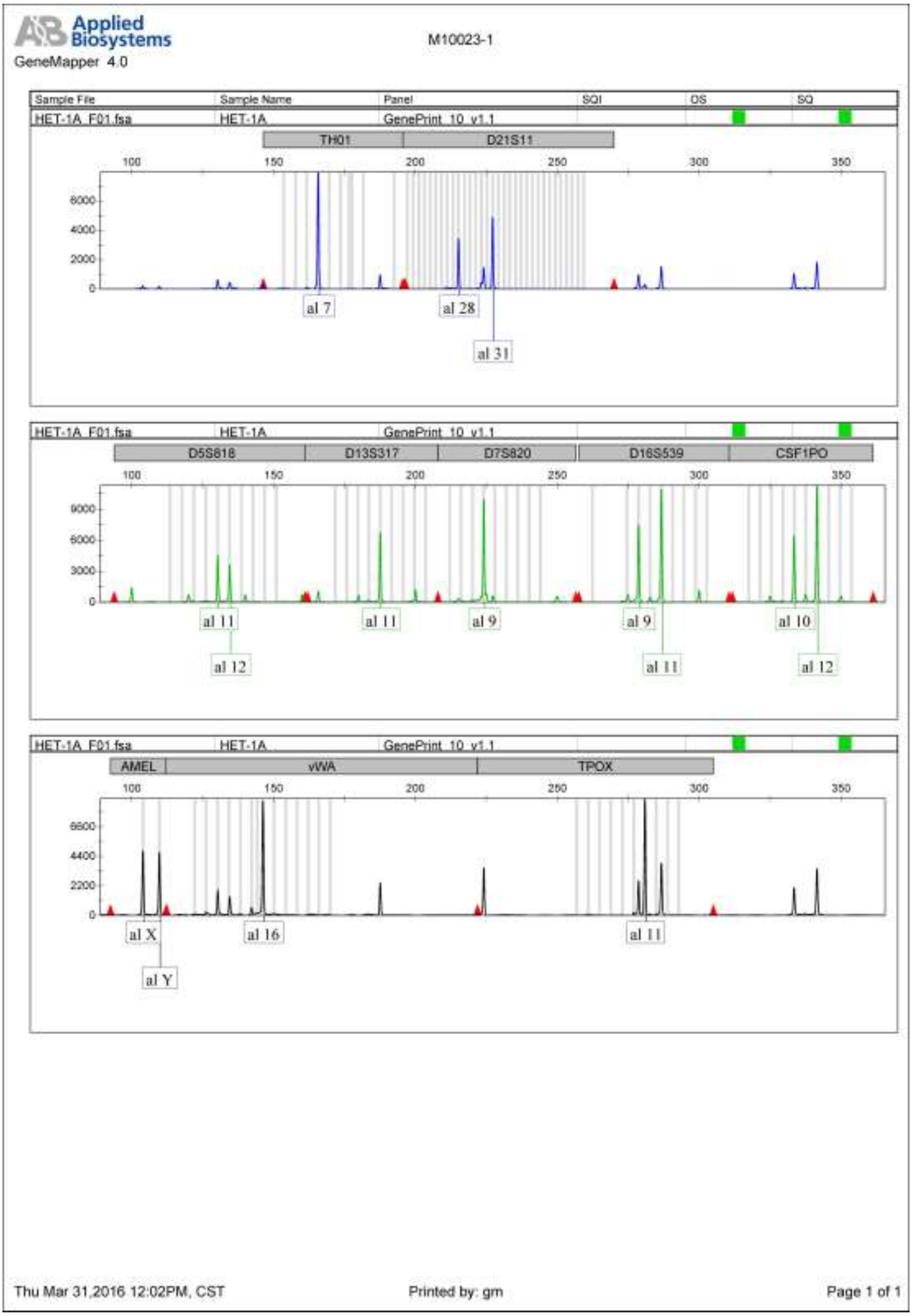

Note：Raw data in appendix
