# Supplementary material for: Ets2 knockdown inhibits tumorigenesis in esophageal squamous cell carcinoma in vivo and in vitro
Source: Oncotarget. 2016 Aug 18;7(38):61458–68. doi: 10.18632/oncotarget.11369 (PMC5308664; doi:10.18632/oncotarget.11369)
Supplement: Supplementary file 5 [file oncotarget-07-61458-s005.pdf]

Ets2 knockdown inhibits tumorigenesis in esophageal squamous cell carcinoma *in vivo* and *in vitro*

Supplementary Materials

Supplementary Material S1: EC1. See_Supplementary_Material_S1

Supplementary Material S2: EC109. See_Supplementary_Material_S2

Supplementary Material S3: EC9706. See_Supplementary_Material_S3

Supplementary Material S4: HET-1A. See_Supplementary_Material_S4
